# Data Report: “Health care of Persons Deprived of Liberty” Course From Brazil's Unified Health System Virtual Learning Environment

**DOI:** 10.3389/fmed.2021.742071

**Published:** 2021-09-20

**Authors:** Janaína Valentim, Eloiza da S. G. Oliveira, Ricardo A. de M. Valentim, Sara Dias-Trindade, Aline de Pinho Dias, Aliete Cunha-Oliveira, Ingridy Barbalho, Felipe Fernandes, Rodrigo Dantas da Silva, Manoel Honorio Romão, César Teixeira, Jorge Henriques

**Affiliations:** ^1^Laboratory of Technological Innovation in Health, Federal University of Rio Grande Do Norte, Natal, Brazil; ^2^Univ Coimbra, Centre for Interdisciplinary Studies of the 20th Century, Coimbra, Portugal; ^3^Multidisciplinary Institute for Human Development With Technologies, State University of Rio de Janeiro (UERJ), Rio de Janeiro, Brazil; ^4^Univ Coimbra, Centre for Interdisciplinary Studies of the 20th Century (CEIS20), Faculty of Arts and Humanities (DHEEAA), Coimbra, Portugal; ^5^Health Sciences Research Unit: Nursing (UICISA: E), School of Nursing of Coimbra (ESEnfC), Coimbra, Portugal; ^6^Univ Coimbra, Centre for Informatics and Systems of the University of Coimbra, Department of Informatics Engineering, Coimbra, Portugal

**Keywords:** sexually transmitted infections, persons deprived of liberty, prisional system, education, SUS—Brazilian national health system

## 1. Introduction

The Brazilian prison system has a history of shortcomings related to lack of investments and infrastructure, leading to severe consequences for the entire prison population, such as problems related to fundamental guarantees of human rights, the lack of health care, and the rise in criminality rates (1–3). With almost the third-largest prison population (4), Brazil accumulates critical issues such as overcrowding, high internal violence indexes, and disease spread (5–7). Data from January to June 2019, from the National Survey of the Penitentiary Information System (Infopen)—Brazil's system of statistical information regarding correctional facilities, published by the National Penitentiary Department (8)—pointed out that the Brazilian prison population was 752,277. Thus, 31,742 people had some condition, 8,523 were HIV-positive, 6,920 had syphilis, 9,113 had tuberculosis, and 7,186 had other diseases.

We present a set of factors that characterize the population deprived of liberty as a vulnerable group to Sexually Transmitted Infections (STIs) (9). Among those that most affect this highly invisible population, Syphilis, HIV, and Tuberculosis are most prominent due to their fast spread and the challenges for diagnosing and accessing treatment (10–12). Yet, in this discouraging spectrum, hope still lingers. It is represented by public policies that genuinely serve such a population and human education aiming to develop its potential fully, including individuals who permeate the prison system in its totality, both those deprived of their liberty and professionals who work there.

Brazilian Prison System, we find such diseases, as mentioned above, quite often. Nevertheless, we believe this scenario can be changed. We believe in a scenario where those conditions can be efficiently avoided and their dissemination rate reduced. We consider that the development of educational resources is a strategy toward human education in health, both for professionals and the community in general. Our investment in education and training strategies is based on studies that indicate the need for educational measures to prevent and promote health care for people deprived of their liberty (13, 14). Taking into account that, besides the high prevalence of these infections in the prison system, we also face knowledge deficits on the subject, misperceptions, and peculiar conditions of imprisonment, which result in at-risk behaviors.

At the core of our observation, the first problem question arises: can a technology-based educational activity train professionals for syphilis and other STIs demands within the prison system? As an answer, we have established the first goal: constructing a data report through an object that combines the two previously mentioned strategies. Thus, (1) a public policy that emphasizes the Virtual Learning Environment of the Unified Health System (AVASUS, in Portuguese: Ambiente Virtual de Aprendizagem do Sistema Único de Saúde) and (2) the course on “Health care of Persons Deprived of Liberty” (15). AVASUS is a virtual learning space for healthcare professionals, students, and general society to enhance SUS training, management, and care. As for the course “Health care of Persons Deprived of Liberty” (ASPPL, in Portuguese: Atenção à Saúde da Pessoa Privada de Liberdade), its learning objectives are characterizing the prison population and introducing the central public policies aimed at this population, with substantial reflections for primary care practitioners. Together, these two resources develop skills that allow for comprehensive care for the person deprived of liberty.

Based on universality, equity and integrity, the ASPPL self-instructional course encompasses the legal and historical contexts of health care of the Brazilian prison population. Its 30-h workload is organized into four units. It focuses on the national prison assistance scenario, the main problems, and specific needs that affect incarcerated people, and the attributions of the Family Health Strategy team—ESF, in Portuguese: Estratégia de Saúde da Família—as to the welcoming and care of people deprived of liberty. The course methodology is based on a proposal of active learning through Problem-Based Learning, simulating real-life situations to stimulate the student's motivation to search for solutions as a starting point for acquiring and integrating new knowledge. In addition to problems, several resources are used, such as texts, animations, interactive timelines, infographics, videos, and games. Thus, it presents a training structure for health professionals to get to know and work in the Brazilian prison system and for the general population to get acquainted with the reality of the penitentiary system, chiefly actions developed in this context.

With the perspective of providing the appropriate content for health professionals and the general population, the course syllabus was produced by a team composed of experts in the field of knowledge and with great practical experience, selected through public notice and submitted to training specific. In the production process, the course was subjected to moments of quality assessment: (a) Technical-scientific assessment; (b) Pedagogical assessment; (c) Brazilian Association of Technical Standards (ABNT, in Portuguese: Associação Brasileira de Normas Técnicas) assessment (standardization) and Portuguese language; (d) Instructional design assessment; (e) Communication assessment; (f) Final evaluation of content writer. AVASUS' pedagogical team accompanies the content writer throughout the course's preparation process until its completion and availability on the platform, ensuring the balance of educational content for access by the target audience.

Then, a second question emerges: with the data of this course participants, is it feasible to measure or map trained professionals for the healthcare demands in the Brazilian prison system in Brazil? The data report we present to answer such question purposes to perform a descriptive analysis of ASPPL's data and provide a repository containing the set of participants' data so that the scientific community may contribute with further research. This repository contributes to the definition of scenarios that enable assessing the quality of health care in the Brazilian prison system and visualizing essential characteristics, such as profile and geographic location, related to the participants who took the course and to the health care facilities. In addition, it allows crossover with epidemiological data on STIs, enabling the analysis of the subject's causal relationship. Finally, the study of this dataset can also help adopt preventive measures that consequently contribute to a decrease in the transmission rate of STIs.

## 2. Materials and Methods

### 2.1. Data Acquisition

The original data of the ASPPL course participants were extracted from AVASUS (15). Since course enrollment is continuously available, data collection comprises from the course's start date to the day of data collection, 06/07/2018 to 05/25/2021, respectively. Initially, the dataset is composed of 14 attributes and 4,861 instances. The attributes, except the unique identifier of the instances (id), correspond to the personal information of the course participants. These are as follows: gender; Brazilian Occupational Classification (CBO, in Portuguese: Classificação Brasileira de Ocupações); participant's occupation through CBO; occupation declared by the participant; course completion percentage; evaluation of the course in free text and on a satisfaction level scale ranging from 1 to 5; geographic location (Municipality and State); employment relationship(s); type(s) of health establishment(s), and whether these are linked or not to the prison system. Attribute types vary, i.e., they are multivalued, and 11 of them have missing values.

### 2.2. Data Processing

Original data was pre-processed through the pipeline described below. In addition, an organized version of the dataset suitable for exploratory data analysis by the scientific community was created. The executed pipeline for data processing, supported by the Python programming language and classical libraries from the data science field, is composed of the following steps: (i) data retrieval and standardization; (ii) treatment of missing data; (iii) data transformation; and (iv) feature selection. The new dataset, formatted as a comma-separated values (.csv) file named “asppl-dataset.csv,” comprises 33 attributes and 4,861 instances. A detailed description of the dataset is available for public consultation at the repository (available at: https://zenodo.org/record/5095518#.YO3gshNueLo).

In the pipeline's first stage, data recovery and standardization (i), work focused on the recovery of missing data on the “gender” and type(s) of health establishment(s) attributes. In the original dataset, 36.65% (1,782) of the instances did not have values referring to participants' gender. After consulting the database of the Permanent Integration System of Education Strategies of the Ministry of Health of Brazil, Sabiá, developed by LAIS/UFRN, approximately 99.44% (1,772) of missing data on the “sex” attribute were retrieved. Continued the recovery process, the instances of the attribute “type(s) of work establishment(s)” to which there was no relevant data were 61.16% retrieved through the National Registry of Health Establishments (CNES, in Portuguese: Cadastro Nacional de Estabelecimentos de Saúde) (16). In standardization, data conventions were created for the following attributes: gender, CBO, State, and employment(s).

For handling the missing data (step ii), missing values of the gender, CBO, and region attributes (see step iii) were replaced. For missing data values of the gender attribute, the term “Not Informed” was assigned, as it was impossible to retrieve the data in step (i) of the pipeline. As per the section 1, the ASPPL course is also open to healthcare students and society in general. That is, anyone, with or without professional registration, can take the course. With this premise in mind, the term “General Population” was assigned to the values absent in the CBO. For missing values for the region attribute, the term “Not Informed” was assigned. The missing values for other attributes were kept to preserve the originality and coherence of instances.

A series of data (pipeline step iii) was replaced and transformed through mappings on secondary sources based on specific attributes from the original dataset. Besides, new relevant attributes were added to the “asppl-dataset.csv” dataset. For the CBO attribute, the numeric code entered by each participant when enrolling in the course was converted into a text that describes the corresponding profession (17). New attributes were also created to store data referring to participants who registered more than one employment and health establishment to avoid loss of information. Furthermore, a new corresponding attribute was created for each employment record (based on CBO) and establishment (based on CNES). According to Brazil's political-administrative and regional division (18) and the attribute referring to the participant's State (Federative Unit, UF), the region attribute was set up. It allows grouping participants into one of the five major regions of Brazil: North, Northeast, Midwest, Southeast, and South.

Conversely, the declared occupation attribute was removed to avoid redundancy and conflicts (pipeline step iv) between official data, registered in the Government database, and informal data. Such data can be consulted through CBO, to which the course participant has also added. Further, to promote more transparency to this pre-processing data stage, we shall indicate the main weakness of the dataset: within the scope of “General Population,” it is possible to create labels, such as “healthcare students” or other occupations. However, due to the technology's limitation, it is not mandatory to declare one's profession.

## 3. Descriptive Analysis

**Figure 1:**
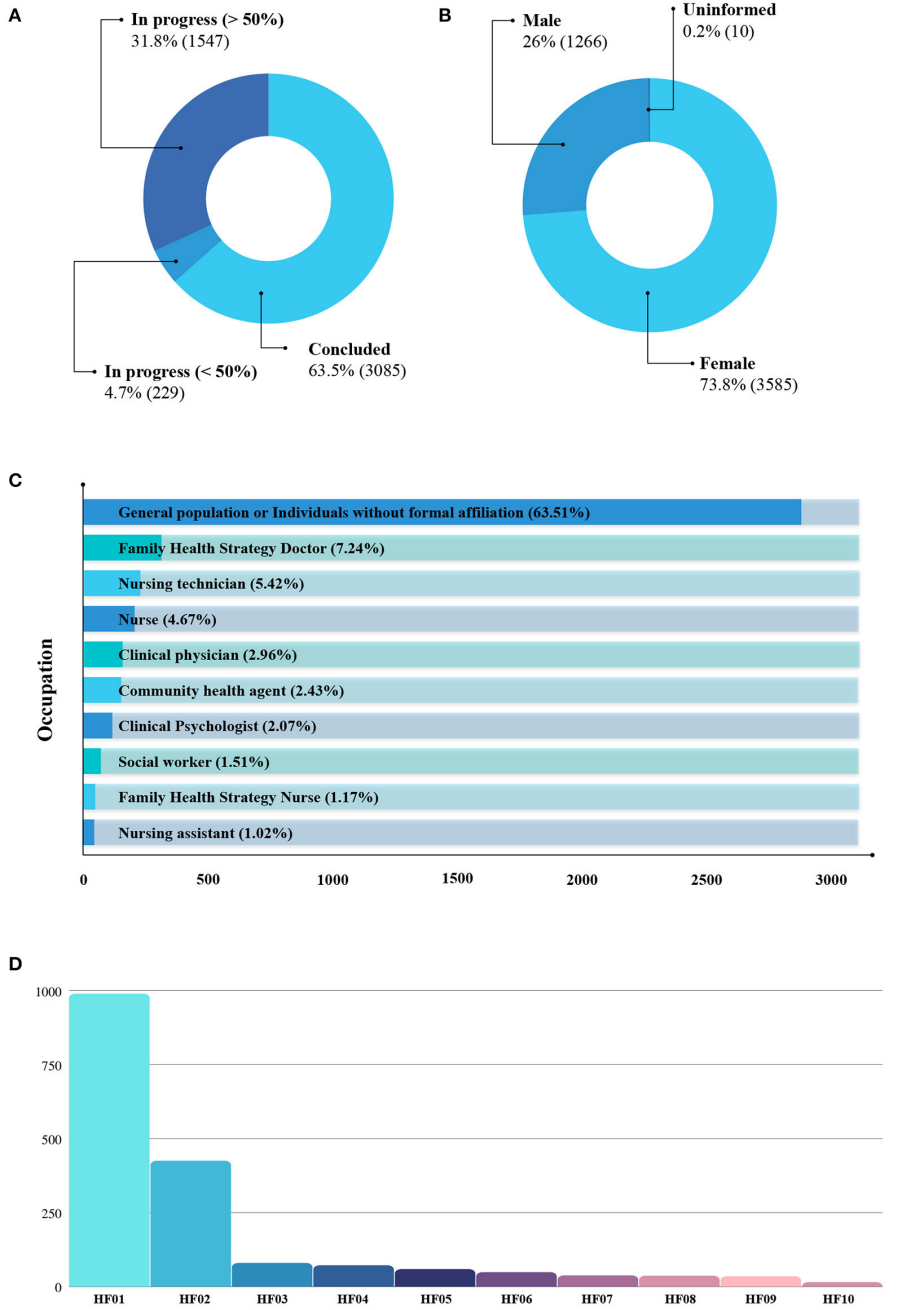
“asppl-dataset.csv” analysis of repository data. **(A)** Number of participants who completed the course. **(B)** Number of participants by Gender. **(C)** Number of occupations of the participants. **(D)** Number of health facilities where participants work. HF01, Health Center/Primary Unit; HF02, General Hospital; HF03, Specialty Clinic/Center; HF04, Health Center; HF05, Health Management Center; HF06, Specialized hospital; HF07, Prompt service; HF08, Polyclinic; HF09, Psychosocial care center; HF10, Isolated office.

The data were analyzed for all 4,861 ASPPL course participants, as available in the “asppl-dataset.csv” dataset, with the aid of the Python programming language. In this pilot study, we mainly analyzed the profile, geographic location, and percentage of course completion of participants. Based on the latter, a total of 3,085 (63.46%) people have completed the course, and 1,776 (36.54%) are currently taking it. Of these, 229 (4.71%) participants attended more than 50% of the program, and 1,547 (31.83%) less than 50%, as depicted in Figure 1A. Out of the group of participants who completed the course, 92.93% (2867) provided an evaluation, ascribing a score from 0 to 5, related to the degree of satisfaction. Therefore, the arithmetic mean of the course evaluations is approximately 4.92, with a 0.38 standard deviation and median equal to 5.0.

Regarding participants' gender, a predominance of females was detected, with a total of 3,585 (73.8%) women participants, as shown in Figure 1B. On the other hand, males are 1,266 (26%) participants. Lastly, those who did not provide gender information and did not have their data retrieved, then called “Not informed,” add up to 10 (0.2%).

We nominally and quantitatively list the first 10 target items to synthesize the occupations and health establishments of the participants who most frequent the course. Figure 1C shows the most frequent occupations. Likewise, we present commercial establishments in Figure 1D. According to the guidelines established by the Ministry of Health, in Brazil, health establishments are categorized by level of care (primary, secondary, and tertiary). Hence, based on these data available in “asppl-dataset.csv,” it is possible to group health establishments and analyze which level of care have trained professionals who meet the health care demands for dealing with STIs in the prison system.

**Figure 2:**
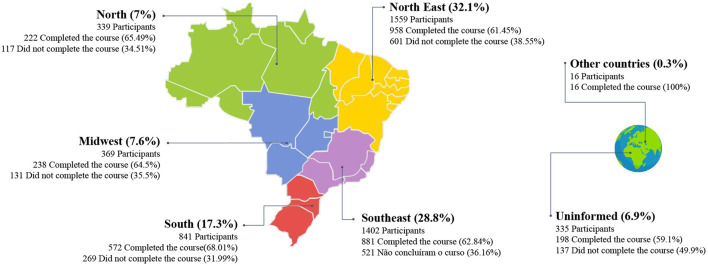
Analysis of participants by region.

For a clearer view of the geographic distribution of course participants, Figure 2 depicts a synthesis of participants by Brazilian region. The course attracted entries from participants from all five of Brazil's regions and their states. In addition to Brazil, it was identified that the ASPPL course had a total of 16 (0.3%) participants residing in other countries. A total of 6.9% (335) of the participants did not inform their place of residence. As for Brazil, it can be observed that the Northeast is the region with the most participants, with a total of 1,559 (32.1%), followed by the Southeast region, with 1,402 (28.8%), South region, 841 (17.3%), Midwest, 369 (7.6%), and the North region, with 339 (7%). Figure 2 reveals the number of participants who completed the course by region.

## Data Availability Statement

The original contributions presented in the study are included in the article/Supplementary Material, further inquiries can be directed to the corresponding author/s.

## Author Contributions

JV, EO, FF, IB, and MR structured and wrote the manuscript. JV, FF, IB, RV, and RS analyzed the data and contributed with descriptive analysis. SD-T, AD, AC-O, CT, and JH reviewed the manuscript. FF, IB, RV, and RS organized the repository. All authors contributed to manuscript revision, read, and approved the submitted version.

## Funding

This work was funded by Ministry of Health Brazil.

## Conflict of Interest

The authors declare that the research was conducted in the absence of any commercial or financial relationships that could be construed as a potential conflict of interest.

## Publisher's Note

All claims expressed in this article are solely those of the authors and do not necessarily represent those of their affiliated organizations, or those of the publisher, the editors and the reviewers. Any product that may be evaluated in this article, or claim that may be made by its manufacturer, is not guaranteed or endorsed by the publisher.
