# Supplementary material for: Data Report: “Health care of Persons Deprived of Liberty” Course From Brazil's Unified Health System Virtual Learning Environment
Source: Front Med (Lausanne). 2021 Sep 20;8:742071. doi: 10.3389/fmed.2021.742071 (PMC8488145; doi:10.3389/fmed.2021.742071)
Supplement: Supplementary file 2 [file Data_Sheet_3.PDF]

Repository

Dataset name: *asppl-dataset.csv*
Version: 1.0
Publication date: MM/DD/YYYY
Dataset period: 06/07/2018- 05/25/2021
Dataset Characteristics: Multivalued
Number of Instances: 4861
Number of Attributes: 33
Missing Values: Yes
Area(s): Health and education

Sources:

- Primary: Unified Health System Virtual Learning Environment (AVASUS, in Portuguese: Ambiente Virtual de Aprendizagem do Sistema Único de Saúde) [1];
- Secondary:
    a. Brazilian Classification of Occupations (CBO, in Portuguese: Classificação Brasileira de Ocupação) [2];
    b. National Registry of Health Establishments (CNES, in Portuguese: Cadastro Nacional de Estabelecimentos de Saúde) [3]; and
    c. Brazilian Institute of Geography and Statistics (IBGE, in Portuguese: Instituto Brasileiro de Geografia e Estatística) [4].

Description: The data contained on the *asppl-dataset.csv* dataset (Table 1) originates from participants of the technology-based educational course “Health care of Persons Deprived of Liberty”. The course is available on the Unified Health System Virtual Learning Environment [1]. This dataset provides elementary data for analyzing the course’s impact and reach, as well as the profile of its participants.

Table 1: Description of Dataset Features.

Attributes	Description	datatype	Value
**Id**	Unique identifier of the course participant (anonymously).	Numerical.	Randomly generated integer.
**Sexo**	Gender of the course participant.	Categorical.	Feminino / Masculino / Não Informado.
**CBO1**	Occupation declared by the participant. Each participant can declare more than one occupation.	Categorical.	Text coded according to the Brazilian Code of Occupations or “População Geral”.
**CBO2**	Occupation declared by the participant. Each participant can declare more than one occupation.	Categorical.	Text coded according to the Brazilian Code of Occupations or NaN.

**CBO3**	Occupation declared by the participant. Each participant can declare more than one occupation.	Categorical.	Text coded according to the Brazilian Code of Occupations or NaN.
**Avaliação do curso**	A score given to the course by the participant.	Numerical.	0, 1, 2, 3, 4, 5 or NaN.
**Texto da Avaliação**	Comment made by the participant about the course.	Categorical.	Free text or NaN.
**Percentual do Curso**	Percentage of completion of the course.	Numerical.	Range from 0 to 100.
**Município**	City where the participant resides.	Categorical.	Name of the city or NaN.
**Estado**	State where the participant resides.	Categorical.	Acronym for the Federative Unit according to IBGE or NaN.
**Total de Vínculos**	Number of employment relationships declared by the participant.	Numerical.	Participant's total jobs or NaN.
**CNES no Sistema Prisional**	Identifies whether the participant's CNES belongs to the prison system.	Boolean	‘True’ or ‘False’
**CNESs**	CNES referring to the establishment where the participant works. Each participant can declare more than one health establishment.	Numerical.	CNES Code or NaN.
**CNESs2**	CNES referring to the establishment where the participant works. Each participant can declare more than one health establishment.	Numerical.	CNES Code or NaN.
**CNESs3**	CNES referring to the establishment where the participant works. Each participant can declare more than one health establishment.	Numerical.	CNES Code or NaN.
**CNESs4**	CNES referring to the establishment where the participant works. Each participant can declare more than one health establishment.	Numerical.	CNES Code or NaN.
**CNESs5**	CNES referring to the establishment where the participant works. Each participant can declare more than one health establishment.	Numerical.	CNES Code or NaN.
**CNESs6**	CNES referring to the establishment where the participant works. Each participant can declare more than one health establishment.	Numerical.	CNES Code or NaN.
**CNESs7**	CNES referring to the establishment where the participant works. Each participant can declare more than one health establishment.	Numerical.	CNES Code or NaN.
**CNESs8**	CNES referring to the establishment where the participant works. Each participant can declare more than one health establishment.	Numerical.	CNES Code or NaN.

**CNESs9**	CNES referring to the establishment where the participant works. Each participant can declare more than one health establishment.	Numerical.	CNES Code or NaN.
**CNESs10**	CNES referring to the establishment where the participant works. Each participant can declare more than one health establishment.	Numerical.	CNES Code or NaN.
**Tipo de Estabelecimento (TE-CNES)**	Identification of the type of establishment the course participant works for.	Categorical.	Text coded according to CNES or NaN.
**TE-CNES2**	Identification of the type of establishment the course participant works for.	Categorical.	Text coded according to CNES or NaN.
**TE-CNES3**	Identification of the type of establishment the course participant works for.	Categorical.	Text coded according to CNES or NaN.
**TE-CNES4**	Identification of the type of establishment the course participant works for.	Categorical.	Text coded according to CNES or NaN.
**TE-CNES5**	Identification of the type of establishment the course participant works for.	Categorical.	Text coded according to CNES or NaN.
**TE-CNES6**	Identification of the type of establishment the course participant works for.	Categorical.	Text coded according to CNES or NaN.
**TE-CNES7**	Identification of the type of establishment the course participant works for.	Categorical.	Text coded according to CNES or NaN.
**TE-CNES8**	Identification of the type of establishment the course participant works for.	Categorical.	Text coded according to CNES or NaN.
**TE-CNES9**	Identification of the type of establishment the course participant works for.	Categorical.	Text coded according to CNES or NaN.
**TE-CNES10**	Identification of the type of establishment the course participant works for.	Categorical.	Text coded according to CNES or NaN.
**Região**	Brazilian region in which the participant resides.	Categorical.	Brazilian region according to IBGE: Norte, Nordeste, Centro-Oeste, Sudeste or Sul.

References

[1] Brasil (2021a). Ambiente virtual de aprendizagem do sus - avasus. atenção à saúde da pessoa privada de liberdade Available from: https://avasus.ufrn.br/local/avasplugin/cursos/curso.php?id=114 .

[2] Brasil (2021b). Classificação brasileira de ocupações - CBO. Available from: http://www.mtecbo.gov.br/cbosite/pages/home.jsf .

[3] Brasil (2021c). Cadastro nacional de estabelecimentos de saúde - CNES. Available from: http://cnes.datasus.gov.br/ .

[4] EducaIBGE (2021). Divisão político-administrativa e regional. Available from: https://educa.ibge.gov.br/jovens/conheca-o-brasil/territorio/18310-divisao-politico-administrativa-e-regional.html .

Article: Data Report: "Health care of Persons Deprived of Liberty" Course from Brazil's Unified Health System Virtual Learning Environment

Authors: Janaína Valentim, Eloiza Oliveira, Ricardo Valentim, Sara Dias-Trindade, Aline Dias, Aliete Oliveira, Ingridy Barbalho, Felipe Fernandes, Rodrigo Silva, Manoel Romão, César Teixeira, Jorge Henriques
